# High-Dose Transarterial Radioembolization of Hepatic Metastases Using Yttrium-90 Resin Microspheres

**DOI:** 10.3390/cancers17243889

**Published:** 2025-12-05

**Authors:** Charlotte C. I. Schneider, Belinda J. de Wit-van der Veen, Sanne M. A. Jansen, Kenneth F. M. Hergaarden, Margot E. T. Tesselaar, Niels F. M. Kok, Larissa W. van Golen, Arthur J. A. T. Braat, Regina G. H. Beets-Tan, Tarik R. Baetens, Elisabeth G. Klompenhouwer

**Affiliations:** 1 Department of Radiology, The Netherlands Cancer Institute, 1066 CX Amsterdam, The Netherlands; 2 GROW School for Oncology and Reproduction, Maastricht University, 6229 ER Maastricht, The Netherlands; 3 Department of Nuclear Medicine, The Netherlands Cancer Institute, 1066 CX Amsterdam, The Netherlands; 4 Department of Gastro-Intestinal Oncology, The Netherlands Cancer Institute, 1066 CX Amsterdam, The Netherlands; 5 Department of Surgical Oncology, The Netherlands Cancer Institute, 1066 CX Amsterdam, The Netherlands; 6 Department of Radiology and Nuclear Medicine, University Medical Center Utrecht, 3584 CX Utrecht, The Netherlands

**Keywords:** radiation segmentectomy, radioembolization, hepatic metastases, yttrium, post-treatment dosimetry, ablative, intra-arterial therapies

## Abstract

**Simple Summary:**

High-dose radioembolization with Yttrium-90 resin microspheres is increasingly used for primary liver cancer, however its role in the treatment of hepatic metastases is unknown. This retrospective study included 15 patients with hepatic metastases and evaluated the safety and efficacy of high-dose radioembolization. The treatment was well tolerated in all patients and disease control after 3 months was achieved in all patients. These findings suggest that high-dose radioembolization is safe and effective for selected patients with hepatic metastases.

**Abstract:**

**Background/Objectives**: Over the past few years, high-dose radioembolization (≥150 Gy) has become widely adopted for the treatment of primary liver cancer, while evidence for its application in hepatic metastases is still limited. The aim of this study was to evaluate the safety and efficacy of high-dose transarterial radioembolization (TARE) in patients with hepatic metastases using resin Yttrium-90 (^90^Y) microspheres. **Methods**: In this retrospective analysis, patients who were treated with high-dose TARE for hepatic metastases with ^90^Y resin microspheres between May 2019 and April 2025 were included. The primary outcomes were treatment efficacy and toxicity assessed according to the National Cancer Institute Common Terminology Criteria for Adverse Events v5.0. Treatment efficacy was evaluated based on radiological response according to Response Evaluation Criteria in Solid Tumors version 1.1, time to progression and overall survival (OS). Secondary outcomes included ^90^Y PET/CT post-treatment voxel-based local deposition model dosimetry and its relations to response. **Results**: A total of 15 patients were included, with hepatic metastases originating from colorectal cancer (*n* = 11, 73.3%), neuroendocrine tumor (*n* = 3, 20%) and breast cancer (*n* = 1, 6.7%). Seven patients (47.7%) had undergone one or multiple prior loco(regional) liver treatments and 13 (86.7%) patients had prior systemic therapy. The median mean tumor absorbed dose was 160.7 Gy (IQR 127.6–245.0 Gy), and the median normal liver parenchyma dose was 40.3 Gy (IQR 21.7–52.3 Gy). Disease control was achieved in all patients, with partial response in 10 patients (66.7%) and stable disease in 5 patients (33.3%) after 3 months. The median OS was 26.5 months (95% CI 24.5 months to no estimate). Two patients (13.3%) experienced grade 3 laboratory toxicity. No grade 4 or 5 toxicities were observed. **Conclusions**: High-dose TARE with ^90^Y resin microspheres resulted in a high disease control rate and demonstrated a favorable safety profile, even in this heavily pretreated cohort.

## 1. Introduction

Hepatic metastases are a common manifestation of advanced malignancies, particularly those originating from colorectal, breast, and other gastrointestinal tumors [1]. Despite advances in systemic therapies, achieving effective locoregional control remains a major therapeutic challenge and key determinant of survival [2].

Transarterial radioembolization (TARE) has emerged as an effective locoregional treatment by delivering targeted β-emitting radiation via yttrium-90 (^90^Y)-labeled microspheres to tumor tissue. The procedure is a multi-step process that involves angiographic mapping, imaging and dosimetry [3]. Pre-treatment simulation with 99mTc-labeled macroaggregated albumin (^99m^Tc-MAA) infusion at predefined catheter positions predicts microsphere distribution over the hepatic metastases and normal parenchyma. Historically, dose planning (e.g., the calculation of radioactivity to be administered) is executed based on empirical models, which are easy to use but often result in suboptimal tumor dosing. The advent of personalized dosimetry and selective infusion techniques has evolved TARE from a salvage therapy with primarily palliative intent into a potentially curative modality for selected patients [3]. These developments enable high-dose TARE, where tumor doses exceed conventional thresholds to maximize the tumoricidal effect while minimizing toxicity.

In hepatocellular carcinoma (HCC), high-dose TARE has demonstrated durable and, in some cases, complete responses [4]. The LEGACY study [5] reported a 88.3% objective response rate (ORR) in patients with solitary unresectable HCC undergoing high-dose TARE, with 27.8% of the patients subsequently undergoing resection or transplantation. The RASER trial [6] showed 90% sustained complete response after radiation segmentectomy (e.g., high-dose radiation to a liver segment). Also, the DOORwaY90 trial [7] confirmed the high efficacy of high-dose TARE in patients with unresectable or unablatable HCC (ORR 98.5%). Based on this evidence, high-dose TARE has been adopted as locoregional treatment in HCC, especially in a curative setting or as down-staging to meet liver transplant criteria [4,8].

In metastatic liver disease, guidelines support the use of TARE for unresectable patients in the salvage setting [2,9]. High-dose TARE has been explored in a limited number of retrospective studies [10,11,12,13,14,15] and is considered a promising treatment option [16]. Here unique challenges are presented due to the infiltrative growth and dispersed tumor distribution, often necessitating treatment of larger perfusion volumes compared to HCC. Clear dose–response thresholds remain undefined, mainly due to the heterogenic microsphere accumulation in metastatic lesions and limited published data on voxel-level post-treatment dosimetry. Furthermore, most high-dose data involve glass microspheres (TheraSphere, Boston Scientific, Malborough, MA, USA) which are due to their higher specific activity more frequently used in this setting; evidence for resin microspheres (SIR-Spheres, Sirtex Medical Europe, Bonn, Germany) remains limited. This study aims to assess the safety, toxicity and clinical outcomes of high-dose TARE using resin microspheres in patients with liver-dominant metastatic disease. Advanced voxel-level post-treatment dosimetry is included to better understand the therapeutic potential in metastatic liver disease.

## 2. Materials and Methods

### 2.1. Participants and Design

This was a single center retrospective study performed under Institutional Review Board approval (IRBd24-003) between May 2019 to April 2025. Patients with liver metastatic disease treated using ^90^Y resin microspheres on clinical indication were eligible for inclusion if they had a planned mean tumor dose ≥ 150 Gy. Exclusion criteria were less than three months follow-up and inability to score response using the Response Evaluation Criteria for Tumor Response (RECIST) version 1.1.

### 2.2. Procedure and Dosimetry Planning

TARE was performed in two phases: planning and treatment phase. During the planning phase, 3D-mapping angiographies (Cone-beam Computed Tomography (CT) or Angio-CT) were performed to identify suitable injection positions and ensure complete tumor coverage. This was followed by intra-arterial injection of ^99m^Tc-MAA and subsequent single-photon emission computed tomography (SPECT/CT). Diagnostic contrast enhanced (CE) CT, 3D-angiographies and ^99m^Tc-MAA SPECT/CT were all used to validate suitability of the patient for TARE according to guidelines.

Individualized dose planning was performed using the partition model. Total liver volume was segmented, as were the treated liver volume and tumor volume per injection position. For uni-or bilobar treatments, the planned mean tumor dose (planTD_mean)_ was maximized (≥150 Gy) while taking into account a <40 Gy threshold to the normal liver in treated volume (<30 Gy for heavily pretreated patients). For segmental treatments, the planTD_mean_ was set at >250 Gy–300 Gy, not taking into account the dose to normal liver in treated volume.

**Figure 1:**
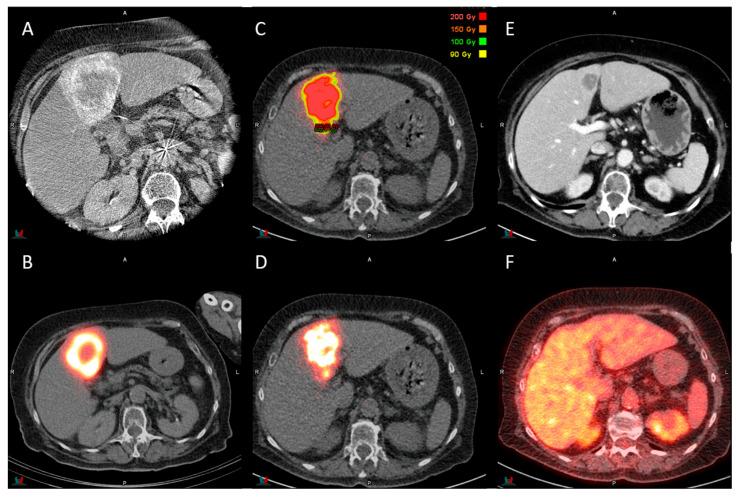
(**A**) Contrast-enhanced cone-beam CT of the treatment volume (segment 4) with one large colorectal metastasis before TARE; (**B**) ^99m^Tc-MAA SPECT/CT used for planning dosimetry showing good ^99m^Tc-MAA uptake in segment 4; (**C**) ^90^Y PET/CT dose maps showing a uniform dose distribution of 200 Gy in the center and a lower dose in the border of the treated volume; (**D**) ^90^Y PET/CT showing good tumor uptake in segment 4 (activity 1.1 GBq; planTD_mean_ 200 Gy; TD_mean_ 259.2 Gy); (**E**) post-treatment contrast-enhanced CT at 3 months showing a remaining lesion (partial response); (**F**) ^18^F-FDG PET/CT at 3 months showing no ^18^FDG uptake in the treated lesion in segment 4.

The treatment phase was performed within two weeks after the initial planning. Patients underwent high-dose TARE with ^90^Y resin microspheres (SIRspheres, Sirtex Medical Europe, Bonn, Germany) administered at planned injection sites, followed by a ^90^Y Positron Emission Tomography (PET)/CT for treatment verification. Figure 1 demonstrates this workflow of planning, treatment and response assessment.

### 2.3. Post-Treatment Dosimetry Analysis

MIM Sureplan^®^ LiverY90 software (version 7.3.6) was used for post-treatment dosimetry. Lung and total liver volume were segmented on the ^90^Y-PET/CT using AI-based contouring. Again, treated liver volume and tumor volume were segmented per injection position on pre- and/or periprocedural CE-CT. The necrotic areas were not removed from the tumor segmentations. Normal liver parenchyma segmentation comprised the treated liver volume excluding the tumor segmentations. Image sets were co-registered with rigid alignment. Tumor volumes < 8mL were excluded from analysis because of partial volume effect [17].

Dose-volume histograms (DVH) and dose statistics of all segmented areas were calculated. The following indices were calculated: (1) the mean absorbed dose (D_mean_); (2) the D70 and D90, to provide the minimum absorbed doses at consecutive percentages within the tumor volume; (3) the V_%<100Gy_, indicating the tumor volume receiving less than 100 Gy; and (4) the AUC_D30–D90_, the area under the curve (AUC) from D30 to D90 calculated using a trapezoidal fit.

A weighted absorbed tumor dose (*wTD*) was calculated per patient, based on a method described by Dimopoulos et al. [18], by summing the product of each tumor’s volume (*V*) and its corresponding dose (*D*), then dividing by the total tumor volume of the patient:

(1) $$Weighted\;Tumor\;Dose\;(wTD)=\;\frac{\left(V_{1}\times D_{1}\right)+\left(V_{2}\times D_{2}\right)+\left(V_{3}\times D_{3}\right)+\cdots }{V_{1}+V_{2}+V_{3}+\cdots }$$

### 2.4. Response and Toxicity Assessment

All available follow-up CE-CTs were reviewed to identify time to progression (TTP) and local response in accordance with RECIST v1.1. On clinical indication ^18^F-FDG PET/CT could be performed; these were not included for response assessment. For colorectal patients carcinoembryonic antigen (CEA) was assessed up to three months after radioembolization, and a change in CEA from baseline was calculated.

Response and progression were assessed in four categories: (1) local response, defined as the targeted lesions within the treated volume; (2) hepatic response, defined as non-targeted hepatic lesions outside the treated volume; (3) extra-hepatic response; and (4) overall response. ORR was assessed at 3 months after TARE for targeted lesions; best response was defined as the optimal radiologic response during the entire follow-up. Disease control rate (DCR) was defined as the proportion of patients achieving complete response (CR), partial response (PR) and stable disease (SD) after TARE. Overall survival (OS) was calculated from moment of TARE to either death or censored at the last recorded follow-up. Biochemical and clinical toxicity were assessed up to 3 months after radioembolization using the Common Terminology Criteria for Adverse Events (CTCAE) version 5.0.

### 2.5. Statistical Analysis

OS and TTP were estimated using the Kaplan–Meier method. Patients were censored at the date of the last hospital visit, in absence of death or progression. All statistical analyses were performed in R v4.4.1 (R Foundation for Statistical Computing, Vienna, Austria). Patient characteristics and dosimetry parameters are provided as median ± interquartile range (IQR) or percentages, when appropriate.

## 3. Results

### 3.1. Demographics and Treatment Planning

A total of 18 patients were eligible for participation in the study; 3 patients had to be excluded due to limited follow-up time or inability to score liver lesions in accordance with RECIST v1.1. Detailed patient characteristics of the 15 included patients are shown in Table 1.

The median planTD_mean_ of the 15 patients was 200 Gy (IQR 187.5–473.0 Gy) based on ^99m^Tc-MAA SPECT/CT. The median treated volume was 1304.7 mL (IQR 646.9–2396.0 mL), with a median tumor volume of 196.0 mL (IQR 87.2–293.0 mL). This resulted in a median administered activity of 2.2 GBq (IQR 1.0–2.9 GBq) ^90^Y microspheres. Administrations were performed as segmental (6 cases), unilobar (5 cases) or bilobar (4 cases) treatments. The total range of pre-calibration days for ^90^Y activity was zero to four days with a median of three (IQR 2–3).

### 3.2. Post-Treatment Dosimetry

**Figure 2:**
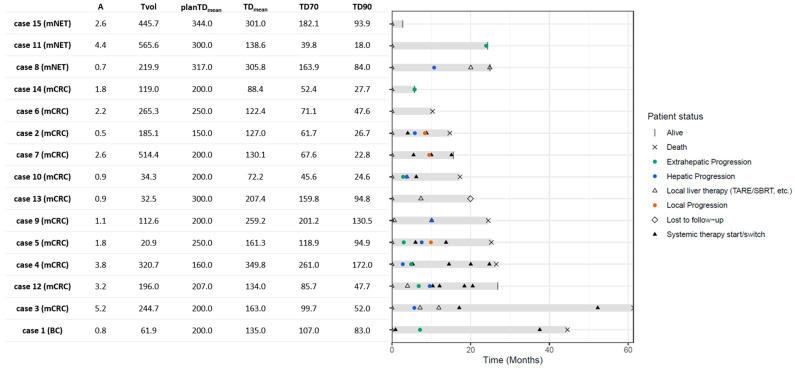
Swimmer’s plot of post-treatment course after high-dose TARE (month = 0) and dosimetry overview per case. A administered activity in GBq; Tvol tumor volume in mL; PlanTD_mean_ mean planned tumor dose in Gy; TD_mean_ mean tumor dose; Dx dose delivered to x% of the volume in Gy.

Based on post-treatment ^90^Y PET/CT, the median TD_mean_ was 160.7 Gy (IQR 127.6–245.0 Gy). The median TD70 was 99.7 Gy (IQR 64.6–161.9 Gy), and the median TD90 was 52 Gy (IQR 27.2–94.3 Gy). The median V_%<100Gy_ was 32.87% (IQR 18.37–53.42%). In Figure 2 and Table S1, the post-treatment dosimetry and course per patient are displayed.

The normal liver parenchyma in the treated volume received 40.3 Gy (IQR 21.7–52.3 Gy); in cases with a uni- or bilobar approach, the median dose was 50.4 Gy (IQR 40.3–87.4 Gy).

### 3.3. Response Assessment

Tumor response at 3 months after treatment is displayed in Table 2. No patients showed progression of treated lesions at 3 months, resulting in a disease control rate of 100%. The best response for local lesions was partial response in 12 patients (80%) and stable disease in 3 patients (20%), with a median time to best response of 7.3 months (IQR 4.9–17.9 months). Complete metabolic response was observed in one patient at 3 months on ^18^F-FDG PET/CT, and in two additional patients during subsequent follow-up scans.

CEA laboratory values were available for eight colorectal patients. All but one patient showed a decrease in CEA within three months after radioembolization (median CEA change: −0.77%, IQR −0.88 to −0.50%).

**Figure 3:**
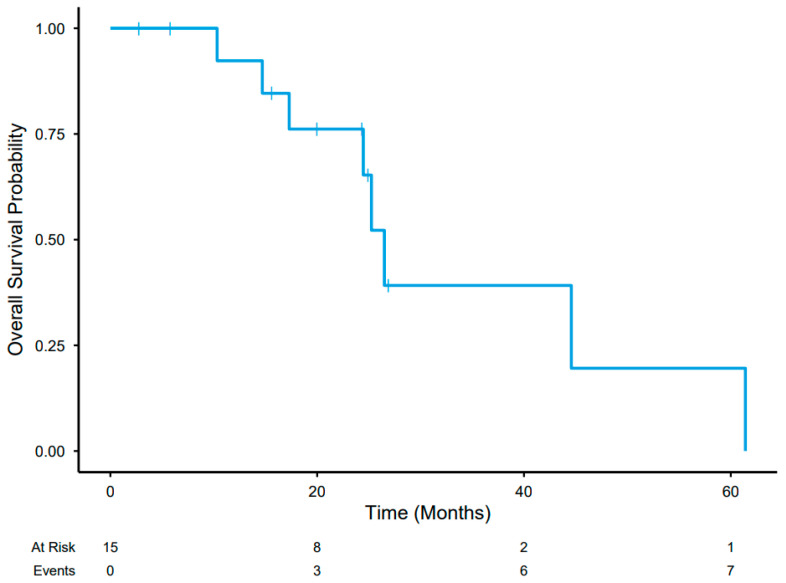
Overall survival in months as estimated by the Kaplan–Meier Method.

The OS was 26.5 months (95% CI 24.5 months to no estimate) for all patients, as shown in Figure 3; OS for the colorectal metastases group alone was 25.2 months (95% CI 17.3 months to no estimate) (Figure S1).

**Figure 4:**
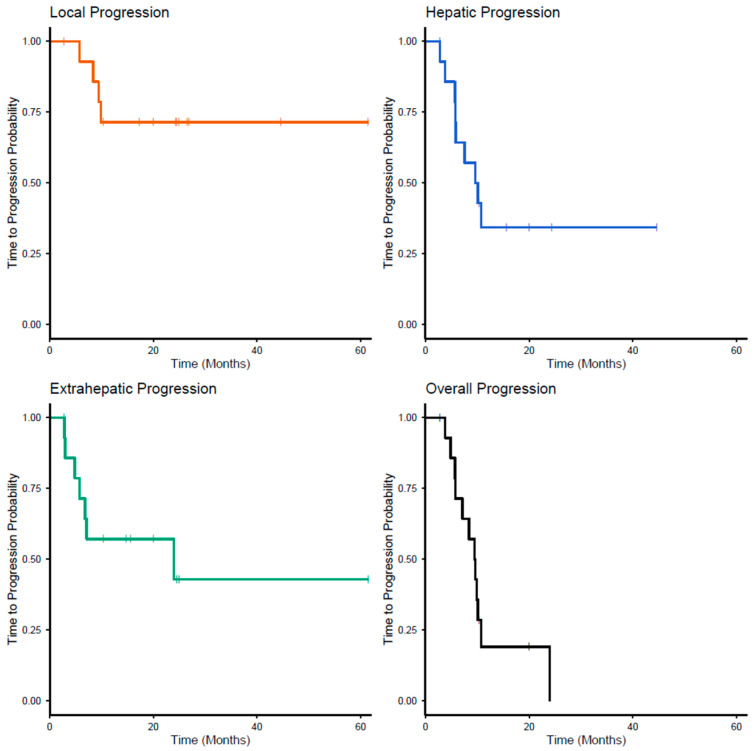
Time to progression (RECIST v1.1 based) as estimated by the Kaplan–Meier Method for local progression, hepatic progression, extra-hepatic progression and overall progression.

The median TTP as assessed by RECIST v1.1 was 9.53 months (95% CI 7.13 months to no estimate), as displayed in Figure 4. Four (26.7%) patients had progression of the local lesions (median TTP was not reached). Nine (60%) patients developed hepatic tumors outside the treated volume, with a median TTP of 9.85 months (95% CI 5.82 months to no estimate). Extra-hepatic progression occurred in seven (46.7%) patients with a median TTP of 23.9 months (95% CI 6.81 months to no estimate).

Patients with local progression had a TD_mean_ of 128.0 Gy (IQR 126.5–161.3 Gy), TD70 of 78.6 Gy (IQR 62.4–98.0 Gy), TD90 of 28.0 Gy (IQR 23.8–46.3 Gy) and V_%<100Gy_ of 46.3% (IQR 31.5–46.6%).

### 3.4. Toxicity

Table 3 displays the toxicity up to three months after TARE. The most frequent grade 1–2 toxicities were elevated aspartate aminotransferase (66.7%), decreased platelet count (40%), anemia (40%), increased alanine aminotransferase (40%) and fatigue (40%). Two patients (14%) experienced a biochemical grade 3 toxicity: elevated GGT in one case and decreased lymphocyte count in the other. No grade 4 or 5 toxicities were observed.

### 3.5. Post-Treatment Course

Most patients experienced extra-hepatic progression before hepatic or local progression, as shown in Figure 2. Eight patients (53.3%) received systemic therapy after radioembolization. Six (40%) patients received one or multiple local liver therapies, consisting of microwave ablation, (repeat) radioembolization, stereotactic radiotherapy and hemihepatectomy. Time after TARE to start a new treatment varied from 1 month to 20 months.

## 4. Discussion

This single-center series demonstrates that high-dose TARE using ^90^Y resin microspheres is feasible, safe and effective for patients with hepatic metastases. Only two patients (13.3%) developed a laboratory grade 3 toxicity. No grade 4–5 events occurred. At the first follow-up, DCR was 100%, and the median OS was 25.2 months. These findings support the use of resin microspheres for high-dose TARE in selected metastatic population, serving as either a bridge to further local therapy or as a break from systemic therapy.

Historically, most high-dose data involve glass microspheres (TheraSphere) due to its higher specific activity (e.g., radiation per microspheres), meaning fewer spheres are needed to deliver a dose. This allows for a higher local tumoricidal doses and more precise dose delivery [3]. The introduction of Sirtex’s FlexDose™ regimen has expanded the capabilities of resin microspheres, allowing physicians to deliver more individualized and higher activity levels [19,20]. It is hypothesized that fewer spheres could prevent vascular embolization, thus reducing the risk of severe complications such as post-embolization syndrome [7,21]. According to the most recent EANM guideline [3], tumor-absorbed doses > 100 Gy have been associated with higher rates of metabolic complete response. In patients with hepatic lesions limited to ≤2 liver segments, higher absorbed doses to the perfused target volume can be administered. For radiation segmentectomies, a tumor-absorbed dose of ≥150 Gy is recommended with ^90^Y resin microspheres in colorectal metastases [3,22]. In the absence of defined thresholds for other tumor types, a planTD_mean_ ≥ 150 Gy has been applied in the current study as the definition for high-dose TARE.

Post-treatment Y^90^ PET/CT imaging revealed a lower tumor-absorbed dose than planned in all but two cases (#4 and #9); although, the current study was not designed to directly compare planning and post-treatment dosimetry. This finding is consistent with prior reports of dose underestimation, even when using voxel-based planning and post-treatment dosimetry [23,24,25,26]. These discrepancies in dose outcomes can be attributed to some fundamental factors: (1) ^99m^Tc-MAA distribution serves as a reasonably good surrogate, but in some patients, hepatic distribution patterns prove to be less predictive; (2) variability in perfusion, flow and catheter positioning between the interventions may cause variations in hepatic distribution patterns; and (3) inherent physical differences between quantification of ^99m^Tc-SPECT versus ^90^Y-PET modalities, which are more pronounced in small lesions [23,24,25]. The unpredictable nature of these factors on microspheres distribution and quantification are not easily accounted for in the clinical setting. Consequently, partition-based planning is still preferred in many institutions over voxel-based planning dosimetry.

Though there is a growing awareness that individualized dosimetry is key in order to optimize treatment efficacy, planning dosimetry is still predominantly reported in clinical studies. As a consequence, there is limited data on the role of post-treatment DVHs and dose statistics for defining dose–response relations. A recent large retrospective study [18] reported improved survival in patients with colorectal metastases for a wTD70 ≥ 60 Gy and wTD_mean_ ≥ 120 Gy. These indices were also calculated using voxel-based dosimetry (MIM SurePlan) based on ^90^Y SPECT/CT or PET/CT scans. To compare, the wTD70 and wTD_mean_ found in this study were higher than these thresholds in all but three cases included in our study (Table S1). Patients with local progression had lower median TD_mean,_ TD70 and TD90 compared to the median of the overall population. Additionally, the local progression group had a higher V_%<100Gy_ compared to the whole population, indicating a higher proportion of tumor volume receiving insufficient dosage [27]. Further research into post-treatment dosimetry across different microspheres and tumor types is essential to establish dose thresholds.

The results of the presented high-dose cohort show favorable results compared to larger registry data on conventional TARE. The CIRT registry [28] reported grade 3–5 toxicity in 11.8% of the colorectal liver metastases (CRLM) patients compared to 13.3% in our cohort. The grade 3–5 toxicity rates of the current series are comparable to prior series describing high-dose TARE ranging from 2.7% to 22% [10,11,12,13,14,15].

In the CIRT registry [29], CRLM patients treated with resin microspheres using the partition model to plan the tumor dose achieved a median OS of 13.0 months (95% CI 8.8–23.8 months). The median OS of 25.2 months in our series exceeds this, though cross-study comparisons should be interpreted with caution because of differences in prescribed activity and treatment strategy.

When compared to high dose studies using ^90^Y glass microspheres [10,11,12,13,14,15], two studies reported an even higher OS of 41.5 months [12] and 60.9 months [11], likely reflecting stricter inclusion criteria. Both studies only included patients with limited hepatic and minimal extra-hepatic disease. The lower overall survival observed in this study may be explained by the high incidence of extra-hepatic disease at baseline. As shown in Figure 4, extra-hepatic progression tended to occur earlier than local and hepatic progression. In patients with neuroendocrine liver metastases, OS exceeding 60 months has been observed [10]. All neuroendocrine patients in our cohort were still alive at the time of data analysis.

Limitations of the current study include its retrospective design, small sample size and heterogeneous study population with multiple primary tumor origins and treatment histories. The small sample size resulted in the inability to perform stratified analyses of the different primary tumor origins. Also, no complete responders were observed in our study based on assessment using CE-CT, whereas others studies have reported complete response following high-dose TARE [10,11,12,13,14,15]. This discrepancy is attributed to the sole use of RECIST v1.1 for response assessment. It is well established that ^18^F-FDG PET/CT provides a more accurate evaluation of treatment response in radioembolization [30], as lesions can remain visible on CT while not having any metabolic tumor activity (Figure 1). As ^18^F-FDG PET/CT was not routinely performed in our institution, it could not be used for standardized response assessment. Despite these limitations, our findings add to growing evidence that high-dose TARE is feasible with resin microspheres and can achieve meaningful disease control and prolonged survival.

## 5. Conclusions

High-dose TARE using ^90^Y resin microspheres is a safe and effective treatment for hepatic metastases from various primary malignancies, even in pretreated patients. Despite variations in clinical characteristics and prior treatments, TARE could provide meaningful clinical outcomes. Future prospective multicenter studies should validate these findings and establish dose–response thresholds for specific cancer types.

## Figures and Tables

**Table 1:** Baseline patient characteristics (*n* = 15).

Variable		Value
Gender	Male	8 (53.3%)
	Female	7 (46.6%)
Age		66 (54.5–71.5)
Primary Tumor Type	Colorectal	11 (73.3%)
	Neuroendocrine	3 (20%)
	Breast	1 (6.7%)
ECOG status	0	9 (60%)
	1	6 (40%)
Prior Systemic Therapy		13 (86.7%)
Prior Local Liver Therapy		7 (47.7%)
Of which *n* patients had	Bland embolization	1 (6.7%)
	Radioembolization	2 (13.3%)
	Surgical resection	3 (20%)
	Chip and burn	3 (20%)
	Ablation (RFA/MWA)	3 (20%)
	Stereotactic Body RT (SBRT)	2 (13.3%)
	Intra-arterial pump chemotherapy	1 (6.7%)
Extra-hepatic disease at baseline		7 (46.7%)
Of which *n* patients had	Lung	6 (40%)
	Lymph node	4 (26.7%)
	Bone	1 (6.67%)
	Soft tissue	1 (6.67%)
Tumor burden (%) ^1^		10.0 (4.7–14.2)
Treatment strategy	Segmental	6 (40%)
	Unilobar	5 (33.3%)
	Bilobar	4 (26.7%)
Baseline lab	Bilirubin, total serum (µmol/L)	6 (6–9)
	Aspartate transaminase (U/L)	28 (24–34)
	Alanine transaminase (U/L	22 (19–28.5)
	Alkaline Phosphatase (U/L)	128 (86.5–201)
	Gamma-glutamyl Transferase (U/L)	98 (48.5–144.5)
Median follow-up time		25.2 (17.3–26.9)

^1^ Tumor burden was calculated by dividing tumor volume (segmented on contrast-enhanced CT) with total liver volume. Data are median (IQR) or n(%). Eastern Cooperative Oncology Group (ECOG); Radiofrequency ablation (RFA); Microwave ablation (MWA); Radiotherapy (RT); Stereotactic radiotherapy (SBRT).

**Table 2:** Local radiological response.

Radiological Response	3 Months Post TARE	Best Response
Complete Response	0	0
Partial Response	10 (66.7%)	12 (80%)
Stable Disease	5 (33.3%)	3 (20%)
Progressive Disease	0	0
Disease Control Rate	15 (100%)	15 (100%)

**Table 3:** Toxicity up to three months after radioembolization assessed according to CTCAE v5.0.

	CTCAE Grade		
Terminology	1	2	3
Abdominal Pain	2	1	0
Anemia	5	1	0
Fatigue	4	2	0
Fever	1	0	0
Nausea	3	3	0
↑ Alanine Aminotransferase	5	1	0
↑ Alkaline Phosphatase	4	1	0
↑ Aspartate Aminotransferase	9	1	0
↑ Blood Bilirubin	1	1	0
↑ Blood Lactate Dehydrogenase	4	0	0
↑ GGT	1	5	1
Hypophosphatemia	1	0	0
↓ Lymphocyte Count	2	0	1
↓ Platelet Count	6	0	0
↓ White Blood Cell Count	1	0	0
Total	49	16	2

↑ indicates increased or hyper-; ↓ indicates decreased or hypo-.

## Data Availability

The used dataset is not available.

## Supplementary Materials

The following supporting information can be downloaded at https://www.mdpi.com/article/10.3390/cancers17243889/s1, Figure S1: Overall survival in months as estimated by the Kaplan–Meier Method for the colorectal metastases population; Table S1: Post-treatment voxel-based local deposition dosimetry per patient.

## Abbreviations

The following abbreviations are used in this manuscript:

^90^Y	Yttrium-90
^99m^Tc-MAA	^99m^Tc-labeled Macroaggregated Albumin
AUC	Area Under the Curve
AUC_D30–D90_	The Area Under Curve Calculated from D30 to D90
CE	Contrast Enhanced
CEA	Carcinoembryonic Antigen
CR	Complete Response
CRLM	Colorectal Liver Metastasis
CT	Computed Tomography
CTCAE	Common Terminology Criteria for Adverse Events
D	Dose
D70	The Mean Absorbed Dose in 70% of the Volume
D90	The Mean Absorbed Dose in 90% of the Volume
DCR	Disease Control Rate
D_mean_	Mean Absorbed Dose
DVH	Dose–Volume Histogram
ECOG	Eastern Cooperative Oncology Group
HCC	Hepatocellular Carcinoma
IQR	Interquartile Range
MWA	Microwave Ablation
ORR	Objective Response Rate
OS	Overall Survival
PD	Progressive Disease
PET	Positron Emission Tomography
planTD_mean_	Planned Mean Tumor Dose
PR	Partial Response
RECIST	Response Evaluation Criteria for Tumor Response
RFA	Radiofrequency Ablation
RT	Radiotherapy
SBRT	Stereotactic Radiotherapy
SD	Stable Disease
SPECT	Single-Photon Emission
TARE	Trans-Arterial Radioembolization
TTP	Time to Progression
V	Volume
V_%<100Gy_	The Percentage of Volume That Receives Less Than 100 Gy
wTD	Weighted Absorbed Tumor Dose
