# Supplementary material for: High-Dose Transarterial Radioembolization of Hepatic Metastases Using Yttrium-90 Resin Microspheres
Source: Cancers (Basel). 2025 Dec 5;17(24):3889. doi: 10.3390/cancers17243889 (PMC12731216; doi:10.3390/cancers17243889)
Supplement: Supplementary file 1 [file cancers-17-03889-s001.zip › cancers-4003389-supplementary.pdf]

Supplementary material

Table S1. Post-treatment voxel-based local deposition dosimetry per patient.

		Tumor										Normal Liver Parenchyma				
**Case**	**Administered dosage(GBq)**	**V (mL)**	**planTD_{mean} (Gy)**	**TD_{mean}**	**wTD_{mean}**	**TD70**	**wTD70**	**TD90**	**WTD90**	**$V_{\%<100Gy}$**	**$AUC_{D30-D90}$**	**V(mL)**	**D_{mean}**	**D70**	**D90**	**$AUC_{D30-D90}$**
**1**	0.8	61.9	200	135.0	135.0	107.0	107.0	83.0	83.0	23.1	6999.9	1399.1	19.8	0.2	0.1	129.2
**2**	0.5	185.1	150	127.0	127.0	61.7	61.7	26.7	26.7	46.6	5243.5	1097.7	8.4	0.1	0.0	10.6
**3**	5.2	244.7	200	163.0	163.0	99.7	99.7	52.0	52.0	30.1	7423.0	2210.5	122.7	40.1	8.1	4790.3
**4**	3.8	320.7	160	349.8	349.8	261.0	261.0	172.0	172.0	2.4	17757.5	2192.2	37.2	0.3	0.1	146.2
**5**	1.8	20.9	250	161.3	161.3	118.9	118.9	94.9	94.9	13.6	8095.9	1957.4	42.6	10.6	2.4	1204.2
**6**	2.2	265.3	250	122.4	122.4	71.1	71.1	47.6	47.6	53.4	5069.1	1315.4	40.3	12.7	4.7	1164.0
**7**	2.6	514.4	200	130.1	145.4	67.6	79.9	22.8	25.2	46.3	5702.7	1884.2	54.3	14.8	5.4	1383.9
**8**	0.7	219.9	317	305.8	356.2	163.9	204.7	84.0	110.3	28.7	14691.7	1756.9	26.8	7.6	2.4	714.7
**9**	1.1	112.6	200	259.2	259.2	201.2	201.2	130.5	130.5	4.3	13412.2	2071.9	16.3	0.2	0.1	16.8
**10**	0.9	34.3	200	72.2	74.1	45.6	47.6	24.6	26.4	77.3	3321.8	1554.7	23.5	0.2	0.1	139.1
**11**	4.4	565.6	300	138.6	138.6	39.8	39.8	18.0	18.0	55.0	4175.3	1900.9	100.0	10.6	0.3	2832.1
**12**	3.2	196.0	207	134.0	134.0	85.7	85.7	47.7	47.7	37.8	6238.4	1550.8	87.4	5.6	0.2	2281.6
**13**	0.9	32.5	300	207.4	207.4	159.8	159.8	94.8	94.8	11.2	10905.5	1329.5	17.1	0.1	0.0	15.4
**14**	1.8	119.0	200	88.4	88.4	52.4	52.4	27.7	27.7	65.8	3928.9	1679.5	50.4	2.6	0.1	1571.4

**15**	2.6	445.7	344	301.0	239.6	182.1	117.5	93.9	56.1	28.6	14369.1	1538.5	50.0	15.6	5.8	1342.9
**Overall (median (IQR))**	2.2 (1.0-2.9)	196.0(87.2 – 293.0)	200 (187.5 - 473.0)	160.7 (127.6 - 245.0)	145.4 (130.4-223.5)	99.7 (64.6-161.9)	99.7(66.4 - 139.4)	52(27.2 - 94.3)	52(27.2 - 95.0)	32.87 (18.4 - 53.4)	6999.936 (5156.3 - 12158.9)	1679.5 (1468.8-1929.2)	40.3 (21.7 - 52.3)	5.6(0.2-11.7)	0.2(0.1-3.6)	1164.0 (134.1-1477.7)

$PlanTD_{mean}$ mean planned dose on tumors; V volume; wTD weighted tumor dose; Dx dose delivered to x% of the volume; $V\%_{0\text{-}100}$ volume percentage that received 0 to 100 Gy; $AUC_{D30\text{-}D90}$ area under the curve calculated based on D30 to D90.

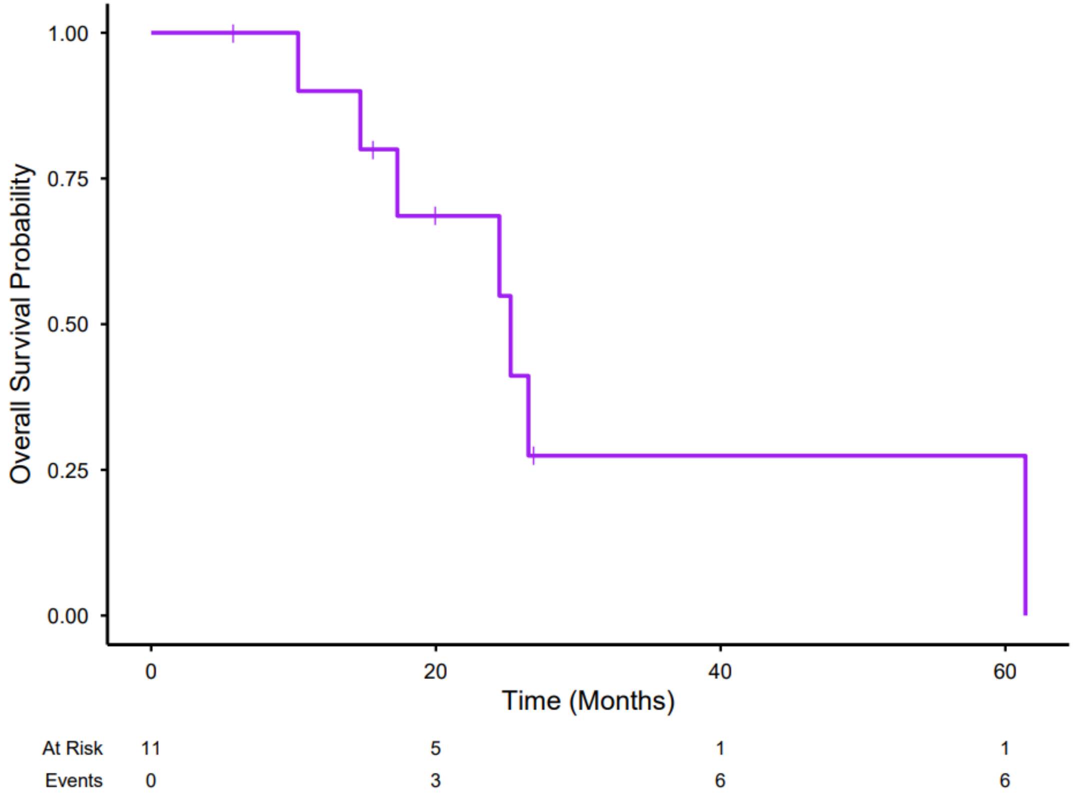

Figure S1. Overall survival in months as estimated by the Kaplan-Meier Method for the colorectal metastases population.
